# Influence of Various Heat Treatments on Hardness and Impact Strength of Uddeholm Balder: Cr-Mo-V-Ni Novel Steel Used for Engine Construction

**DOI:** 10.3390/ma14174943

**Published:** 2021-08-30

**Authors:** Paweł Mazuro, Julia Pieńkowska, Ewa Rostek

**Affiliations:** 1 Department of Aircraft Engines, Faculty of Power and Aeronautical Engineering, Warsaw University of Technology, 00-661 Warsaw, Poland; pmazuro@itc.pw.edu.pl; 2 Centre for Material Testing and Mechatronics, Motor Transport Institute, 03-301 Warsaw, Poland; ewa.rostek@its.waw.pl

**Keywords:** impact toughness, hardness, CrMoVNi steel, Charpy impact test, heat treatment, quenching, tempering

## Abstract

The construction of an engine requires optimized geometry and superb material properties in various environments. Tensile and yield strength are not the only parameters essential to consider. Hardness, impact toughness, and ductile-brittle transition temperature (DBTT) are also crucial. In this paper, Balder, Chromium-Molybdenum-Vanadium-Nickel steel with low impact toughness attested is considered. It contains both high Nickel and high Vanadium content, a rare combination among iron-based alloys. This study aims at proving that conventional heat treatment can improve its impact toughness while maintaining hardness level, exceeding its to-date performance. Steel’s exact elemental composition was checked, and material samples’ hardness and impact toughness were measured. Four heat treatments were proposed, then hardness and impact toughness were measured again. It was established that impact toughness over three times higher than marketed (57.3 J against 17 J) can be achieved with simultaneous 2 HRC points (from 46.4 HRC to 48.4 HRC) rise in hardness. Achieved parameters place examined alloy at the high-ranking position among similar steels. Occurrence of temper embrittlement was avoided. Notably, the ductile-brittle transition was not observed in any sample.

## 1. Introduction

Despite being around for over a century, steel alloys still play a major part in construction and engineering. With more advanced applications grows the demand for specialized, high-performing materials. Since the industrial revolution, experiments with alloying elements have been conducted with good results [1,2]. Pre-WWII metallurgy already saw alloys containing multiple additions, enabling record-breaking engine performance. As an example, Junkers Jumo 205 had connecting rods made with steel containing 0.35% C, 0.2% Si, 0.34% Mn, 0.03% S, 0.027% P, 3.84% Ni, 1.14% Cr, 0.03% Mo and 0.04% V. Complex alloying paid off; combustion pressure exceeded 100 bar, rising with over 10 bar/s, without con-rods failure [3]. The relatively high Nickel content is worth noting, as the addition of up to 4 wt. % provides the best results in increasing impact toughness [4].

Much work has been made to determine the influence of elements such as Nickel [5,6], Molybdenum [7,8], Vanadium [9], and Chromium [10] on steel behavior. Various amounts and combinations of those elements have been studied in detail before. However, iron-based alloys with simultaneously high Nickel and Vanadium content are rare.

The effect of impurities has also been studied [8,10,11], and methods of purifying steel alloys have been devised, such as Electro-Slag Remelting (ESR) or Vacuum Arc Remelting [10].

Phenomena governing metals’ behavior, such as temper brittleness, became increasingly possible to study. Since the early studies [12,13] with their limitations, temper embrittlement (TE) and correlated Ductile-Brittle Transition Temperature (DBTT) were examined in increasing detail from multiple angles [7,11,14,15,16]. New techniques were developed along the way, such as magnetic nondestructive evaluation [17].

Low alloy steels with varying Ni, Cr, Mo, and V additions remain a valuable research topic, despite this metal group’s long history, as they play an essential role across engineering applications. Their good mechanical properties are valued in shipbuilding and aerospace manufacturing, among other fields [18]. Continued demand prompts continued development by manufacturers and should be followed by continued research of newly produced alloys. Such studies bring several benefits, helping to organize existing metallurgical knowledge and shedding light on new materials. Treatments alternative to the manufacturer’s guide can lead to significant mechanical properties’ improvements, thus enabling a given alloy to be used in a broader range of applications.

In this paper, Balder, Chromium-Molybdenum-Vanadium-Nickel steel is considered. The producer describes it as having excellent high-temperature strength, great machinability and is suitable for nitriding and nitrocarburizing [19]. Those statements and chemical composition are the basis on which steels for comparison were picked.

It has a unique chemical composition, as it is rare for steel alloys to have high Vanadium and high Nickel content. Literature [20,21,22,23,24,25,26,27,28,29,30] shows comparable hot work steels with Vanadium content higher than 0.4 wt. % and only have small amounts of Nickel in them (up to 0.35 wt. %).

Data available on heat treatment states that it is delivered pre-hardened to the range of 42–45 HRC when tempered at 590 °C 2 × 2 h [31]. Graphs in [19] show that tempering parameters described above pull material just past the temper embrittlement phase. According to said graphs, steel should exhibit a hardness of around 46 HRC and impact strength of around 16 J. It is noteworthy that the producer’s data shows this alloy’s impact toughness is almost as low as it could be. Temper embrittlement is mentioned as an explanation; however, it remains unclear to authors why such a decision was made, and the “basic” as-delivered variant was not improved.

The steel of similar constitution proved to be an excellent material for parts working in harsh conditions in an engine, capable of reaching about 40 J of impact strength [32], provided proper heat treatment has been used. Several similar alloys were chosen for comparison, proving it not to be an isolated case [20,21,22,23,24,25,26,27,28,29,30,31]. It is shown that for steel of around 46 HRC, impact toughness substantially higher than 16 J can be obtained.

As high Nickel content effectively moves DBTT into negative temperatures [5], its occurrence at −29 °C is unlikely. Charpy impact test in that temperature will be conducted to confirm this. Steels of the aforementioned compositions are rare, and research is lacking. This study, however, aims to improve Balder’s mechanical properties through conventional heat treatment, therefore shedding light on its potential and serving as an introductory guide for further research directions.

## 2. Materials and Methods

The as-delivered alloy was subjected to optical emission spectrometry analysis to ensure the producer’s chemical composition matches.

For this analysis, spark-optical emission spectrometer SPECTROMAXx (SPECTRO Analytical Instruments GmbH, Kleve, Germany) with argon shield atmosphere, equipped with iCAL calibration logic, was used. It allows us to conduct both qualitative and quantitative analyses of ferrous and non-ferrous alloys. Via testing, contents of 54 elements used in metallurgy can be obtained. In the pre-spark phase, it can detect free graphite and automatically point out bad samples.

Results of four samples are presented in Table 1. Element contents mentioned in obtained producer’s data are listed in column “typical”. Contents of other elements (e.g., Sn, Ca) were less than 0.01 wt. % and are summarized in row “Other”.

Element contents are within a 10% margin, compared to available data for all elements except silicon, which is 43% higher than typical. Si is known to influence machinability negatively and increase brittleness. Such deviation from declared value shows the importance of verification of chemical composition ahead of planned testing; however, in this particular case, a significant decrease in an alloy’s mechanical properties is not expected due to its overall small amount. What does, on the other hand, make a difference in machinability is Vanadium content. V decreases it significantly more than most alloying elements, which is why such a high amount of it is somewhat surprising in an alloy described as having “superior machinability” [19]. Those doubts were confirmed during machining; cutting inserts showed significantly shorter lifespan working on Balder, compared to similar details from 40CrMnNiMo8-6-4 steel. It suggests machinability may not be this alloy’s biggest strength, and its other qualities should be emphasized over it.

All Chromium, Molybdenum, and Vanadium are used to homogenize the structure of an alloy, reduce grain size, and increase heat resistance. As they all tend to form carbides [33], under certain circumstances, especially in high alloy steels, secondary hardening may occur (increasing hardness during tempering). This possibility will be evaluated later.

The amount of Nickel seems to be carefully considered in terms of impact toughness since the addition of 2.7 to 4 wt. % of it proved to have the best results in increasing that parameter [4].

Content of impurities (P and S) is much higher than levels achievable in modern metallurgy. Negligence regarding the purity of steel can at times bear serious consequences. For example, some alloying elements (especially Phosphorus) may impact temper embrittlement, as explained later. The above makes purifying an alloy worth considering.

Overall, the presented steel has a promising chemical composition, comparable to other hot-work steels. It seems to be able to provide satisfying mechanical properties after considerate heat treatment.

### 2.1. Hardness Measurement

Rockwell C hardness test was conducted using the Łucznik PW106 machine (Łucznik, Radom, Poland). Procedures complied with PN-EN ISO 6507-1:1999 standard [34]. Four samples at room temperature were tested three times each. Measurement points were at least 6 mm apart. Results will be presented with corresponding impact test data for more straightforward analysis.

### 2.2. Impact Toughness Test

Charpy impact test was conducted to determine whether obtained material shows the same properties as declared by the producer. Samples were tested using INSTRON impact test machine with Dynatup 9250 HV drop weight column (INSTRON, Bristol, UK). It enables adjusting impact energy in the range of 4.6–945 J by manipulating mass and height of weight drop. Velocity sensors’ accuracy is +/−0.25% in the range up to 12 m/s. An optical encoder, which is also a part of the setup, can measure displacement with +/−0.05% accuracy. The expanded uncertainty of the impact energy measurement equals ±6.5 J (p~95%, k = 2). The machine is equipped with the Impulse data acquisition module, allowing rapid load data collection during test and data post-processing (charts, tables, comparisons with other test results).

**Figure 1:**
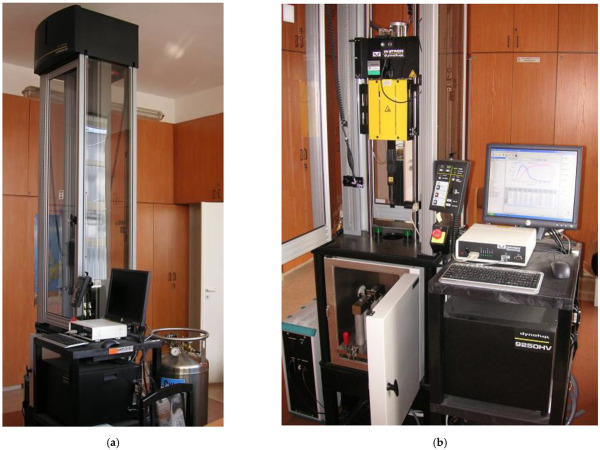
(**a**,**b**) Test setup for Charpy impact test.

Test setup with data acquisition module is shown in Figure 1a,b.

**Figure 2:**
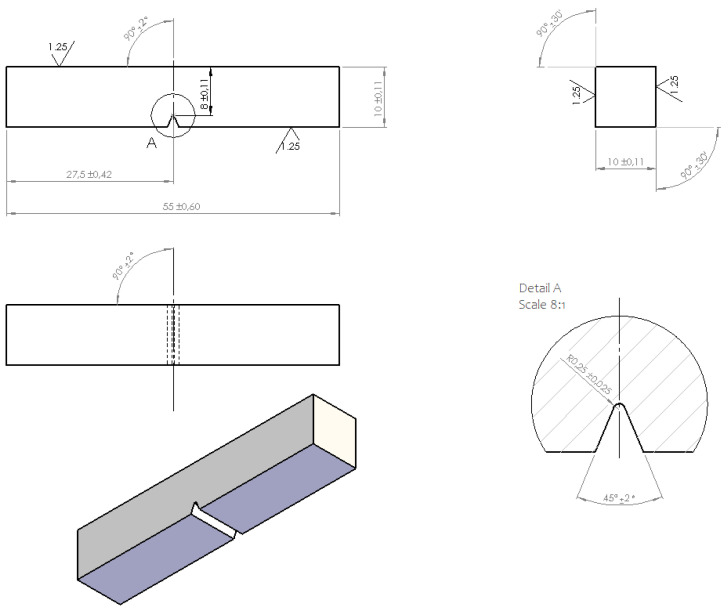
Charpy V-notch test sample geometry (dimensions in mm).

V-notch samples were prepared as specified in [35]. Detailed sample geometry is shown in Figure 2.

Procedures complied with PN-EN ISO 148-1:2017-02 standard [35], and a test was performed on standard-size samples specified in the said norm. The energy exerted on a sample was about 300 J with a drop velocity of ~5.3 m/s. Tests were conducted in 20 °C ± 2 °C (samples 1 and 2) and −29 °C ± 2 °C (samples 3 and 4). Ambient temperature was controlled using a LAB-EL thermohydrometer (LB-700 type) (LAB-EL, Reguły, Poland). Sample temperature was measured using a LAB-EL digital thermometer (LB-522TX type) with an external TA-TP sensor (LAB-EL, Reguły, Poland).

Graphs showing loads (blue lines, in kN) and impact energy (red lines, in J) in time (in ms) and corresponding fracture area pictures are presented in Table 2.

A lack of apparent yield strength was observed in samples during tests, contrary to the manufacturer’s data, where yield tensile strength equals 1230 MPa for 44 HRC variant and 1320 MPa for 47 HRC variant. Selected data is presented along with hardness measurements in Table 3.

**Figure 3:**
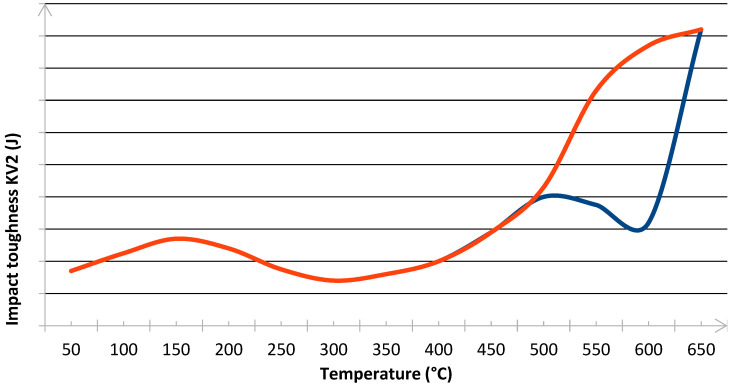
Impact toughness vs. tempering temperature in fast (orange line) and slow (blue line) cooling scenarios.

Pictures show that samples made from as-delivered material tend to fracture bristly (only a little deflected area is visible). The fracture surface resembles that of high hardness (50 HRC and more) tool steel. Considering that the hardness of steel in question is noticeably lower, it confirms temper embrittlement. It is known that during tempering at around 550 °C ± 50 °C if the cooling rate is too low, the impact toughness of many steel alloys drops instead of rising steeply with temperature. A qualitative depiction of a relationship between impact toughness and the cooling rate is presented in Figure 3. The blue line represents slow cooling, while the orange line represents fast cooling.

It is believed that the drop in impact toughness in those conditions is not solely related to the decomposition of retained austenite but also to the concentration of Phosphorus on grain boundaries, which is considered to be the main temper embrittlement-causing impurity [36,37,38]. Some alloying elements such as Titanium, Niobium, Molybdenum, and Vanadium show efficacy in mitigating Phosphorus concentration on grain boundary [8]. It is suspected that a few mechanisms contribute to that, such as Ti and Nb “pinning” P inside of the matrix or settling on the grain boundary themselves, thus restricting the amount of space Phosphorus can take up [39]. However, the exact effects are difficult to quantify, especially since all those elements show the ability to form carbides [33], limiting their anti-embrittlement effect. Molybdenum additions’ impact on inhibition of scavenging (segregation to the grain boundary) of Phosphorus is unclear, as studies [40,41,42] reach conflicting results. The same can be said about Vanadium, as it does, to some extent, mitigate the effect of Phosphorus, but the exact mechanisms are yet to be described.

With this number of overlapping effects in play, it is incredibly challenging to determine a “safe” amount of Phosphorus in respect of temper embrittlement [39]. Thus, restricting the overall number of impurities seems to be a better solution, especially nowadays, with modern advances in metallurgy.

In general, temper embrittlement can be prevented in two ways; by changing the chemical composition of an alloy (primarily eliminating an extensive amount of Phosphorus and other impurities) or adjusting heat treatment, with particular attention being paid to cooling rate. This research focuses on improving the properties of an alloy of set composition; thus, metallurgical work will be omitted, and only hardening methods will be considered.

Data regarding samples’ dimensions, hardness, and impact toughness are presented in the Table 3. As mentioned before, the expanded uncertainty of the impact energy measurement equals ±6.5 J (p~95%, k = 2).

For comparison, data on hardness and impact toughness of similar ferrous alloys is presented in Table 4. Their chemical composition is shown in the Table 5.

Data in Table 4 concludes that better decisions regarding pre-hardening could have been made on the production stage of examined alloy. The producer’s brochure [19] states that this steel can be used without additional heat treatment. However, the authors would advise against this.

A range of hot work steels similar to Balder is presented in the Table 5. As mentioned before, simultaneously high Nickel and Vanadium contents are hard to come by. Among presented comparable steels, only 30HN2MFA has non-negligible contents of both of them.

### 2.3. Heat Treatments

Four heat treatments (HT) were proposed. After each treatment, hardness and impact toughness were measured at 20 and −29 °C, complying with standards mentioned before. For more reliable results, two samples were tested in each time-temperature combination, giving a total of 16 experiments. Sample number, exact heat treatment it was subjected to, and temperature of the impact test sample (Sample Temperature, ST) are listed in the Table 6.

Both quenching and tempering were conducted in oil. While water and particularly aqueous solutions of inorganic salts provide a very high cooling rate, it comes at the cost of a high risk of cracking. Quenching in oil is much safer in that regard. However less extreme than cooling in water, it can still act as a sort of fuse; if a part quenched in oil will survive the process undamaged, subsequent stages of heat treatment will likely cause it no harm as well. This approach can prove especially useful when multi-stage treatment of a part is necessary. Detecting mistakes early on is a cost-saving measure in such cases.

Due to a limited number of heat treatments proposed and significant differences between them, it cannot be stated that any of them will provide an optimal hardness-ductility ratio for this alloy. Processes were chosen based on the authors’ previous experiences with treating similar steel. As this alloy shows a relatively high content of impurities, it was reasonable in the authors’ view to first try more straightforward, conventional heat treatments. If significant improvements are made, research into more sophisticated methods, such as cryogenic treatment, will be well-based.

## 3. Results

After heat treatment hardness of each sample was measured, measurements were conducted as described earlier, and results, as previously, will be listed with corresponding impact test data.

Charpy impact test was performed using the same setup as in preliminary tests on as-delivered samples. Graphs depicting energy absorbed by the sample (red lines, in J), the load exerted (blue lines, in kN) in time (in ms), and the fracture area picture for each experiment are listed in the Table 7. Impact toughness extracted from tests, and hardness measured before a sample was destroyed, are shown in the Table 8.

Throughout the experiments, rise of ductility in comparison with as-delivered samples is easily noticeable. A higher amount of energy absorbed corresponds to the amount of section area shear lips take up, as they consume about half of the energy of ductile fracture [43].

Data on mean impact energy absorbed and mean hardness each sample pair exhibits are presented in Table 8. As mentioned earlier, expanded uncertainty of the impact energy measurement equals ±6.5 J (p~95%, k = 2). Yield strength and tensile strength were not measured separately but are approximated based on hardness. Pavlina and Van Tyne’s research shows a linear correlation for HV > 130 [44]. Relationship between Vickers hardness (Hv, [kgf/mm²]) and yield strength (YS, [MPa]) is characterized by Equation (1), and its relationship with tensile strength (TS, [Mpa]) by Equation (2). Hardness in HRC was converted to HV based on information in [22].

YS = −90.7 + 2.876 Hv(1)

TS = −99.8 + 3.374 Hv(2)

**Figure 4:**
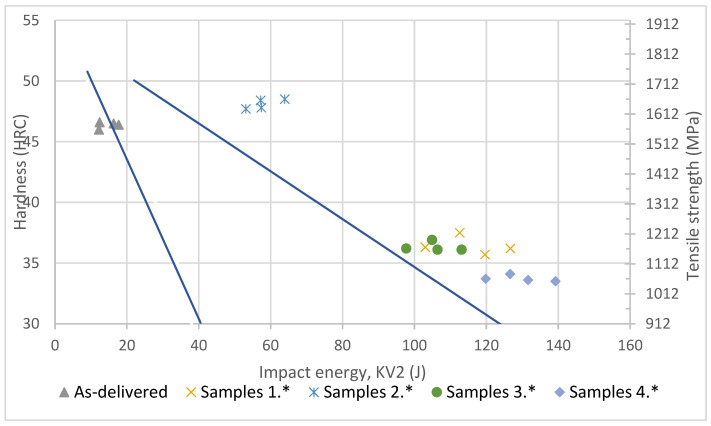
Comparison of pre- and post-treatment samples against similar steels’ properties.

Experimental data for each sample are presented in Figure 4 Dark blue lines represent the typical range of parameters of similar hot work steels. They were drawn based on the authors’ previous research, which is not the subject of the present paper. The closer to the bottom left side of the graph, the lower the quality of an alloy. Samples after additional heat treatments proposed in this experiment present a better ratio of mechanical parameters than as-delivered samples. Further optimization of heat treatment procedures is likely to yield even better results.

## 4. Discussion

The data obtained through the experiment show significant improvement in the mechanical properties of the alloy in question. Excellent results were yielded through treatment 2. (quenching in 960 °C for 25 min, tempering in 200 °C 2 × 2 h). The temperature of the process was set lower than the range in which temper embrittlement occurs; thus, it was avoided. According to the graph in [19], Balder presents impact toughness of around 40 J when tempered at 200 °C for 2 × 2 h. An increase in alloy’s ductility through this treatment is attributed to the time-temperature combination of the quenching period. Authors believe it proved more efficient than the manufacturer’s process in mitigating grain size growth, commonly known to decrease impact toughness [45].

Charpy impact test in −29 °C has shown no significant decrease in impact toughness against the room temperature test. It was suspected due to the high Nickel content [5]. Grain refinement may serve as further explanation, as it would be in line with Hall-Petch law regarding DBTT.

As an example of steel alloys rich simultaneously in Nickel and Vanadium, Balder proved to be a promising material to explore and should be studied further. Microstructure examination could provide valuable data regarding the influence of Nickel in the presence of high Vanadium content in steel alloys. As Phosphorus content is relatively high, and Balder shows vulnerability to temper embrittlement in certain conditions, it can help us to better understand this phenomenon. Properties obtained through conventional heat treatments are promising compared with a range of hot-works steels, and authors recommend research into more sophisticated methods, such as cryogenic treatment.

From the manufacturer’s point of view, Balder exhibits properties promising enough to justify more attention to the purification of this alloy.

## 5. Conclusions

In this paper, Balder, Chromium-Molybdenum-Vanadium-Nickel steel was considered. After its composition and marketed properties were confirmed, it was subjected to four different heat treatments to determine if its impact toughness can be improved with little sacrifice to hardness. Conclusions are as follows:

(1) Occurrence of temper embrittlement was confirmed, and possible explanations were proposed. Authors suspect it originated in a cooling rate lower than critical, and additionally, non-negligible content of Phosphorus segregating to the grain boundary contributed to that phenomenon. Thus, to avoid it, either purifying an alloy or more thought-through heat treatment should be considered.
(2) Tests conducted after heat treatment of samples show substantial improvement in mechanical properties of the examined alloy. Mainly, an increase in hardness from 46.4 HRC to 48.4 HRC with impact toughness simultaneously rising three-fold (from 17 J to 57.3 J) was recorded, and a six-fold increase of impact toughness (from 17 J to 135.4 J) was possible with a hardness drop from 46.4 HRC to 33.5 HRC. It proves that striking a satisfying balance between those two properties is indeed possible.
(3) Ductile-brittle transition was not detected in temperatures as low as −29 °C, making alloy in question a promising material for use in engine applications.
(4) Secondary hardening was not observed; as-delivered material decreased in hardness when tempered.

## Figures and Tables

**Table 1:** Chemical composition of an alloy.

Element	Sample 1.	Sample 2.	Sample 3.	Sample 4.	Average	Typical
C (%)	0.33	0.33	0.33	0.33	0.33	0.3
Si (%)	0.43	0.43	0.43	0.43	0.43	0.3
Mn (%)	1.14	1.13	1.13	1.14	1.14	1.2
Cr (%)	2.34	2.34	2.34	2.34	2.34	2.3
Mo (%)	0.78	0.78	0.78	0.78	0.78	0.8
V (%)	0.83	0.81	0.82	0.81	0.82	0.8
Ni (%)	3.88	3.88	3.88	3.88	3.88	4.0
P (%)	0.014	0.014	0.014	0.014	0.014	-
S (%)	0.029	0.021	0.027	0.03	0.027	-
Al (%)	0.019	0.021	0.02	0.02	0.02	-
Co (%)	0.019	0.019	0.02	0.02	0.02	-
Cu (%)	0.14	0.094	0.074	0.066	0.093	-
W (%)	0.026	0.025	0.025	0.025	0.025	-
Te (%)	0.021	0.021	0.021	0.021	0.021	-
Ta (%)	0.074	0.074	0.077	0.065	0.072	-
B (%)	0.0007	0.0006	0.0005	0.0007	0.0006	-
Fe (%)	89.8	89.9	89.9	89.9	89.9	Bal.
Other (%)	Bal.					-

**Table 2:** Test results—Fracture pictures and graphs of exerted energy and load (as delivered material).

Sample No.	Graph	Fracture Area
1	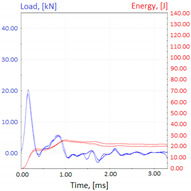	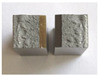
2		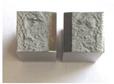
3	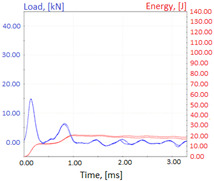	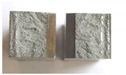
4		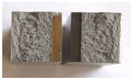

**Table 3:** Samples’ dimensions and tests results.

Sample Number	Sample Width (mm)	Notch Bottom Position (mm)	Maximum Load (kN)	Impact Energy KV2 (J)	Mean Impact Energy KV2avg (J)	Mean Sample Hardness (HRC)	Average Material Hardness (HRC)
1	9.97	8.00	18.94	16.3	17.0	46.5	46.4
2	9.96	7.99	20.25	17.7		46.4	
3	9.97	8.00	14.86	12.2	12.3	46	
4	9.97	8.00	14.91	12.4		46.6	

**Table 4:** Comparable hot work steels’ parameters.

Steel Alloy	Standard Hardness (HRC)	Standard Impact Toughness KV (J)	Source
H13	46 38–53	~26 Very high	[20] [21]
33H3MF	26	92	[22]
Orvar Supreme	45	~16	[23]
WCLV	56	25.6	[24]
	48	32	
25H2N4WA	27.6	63	[25]
30HN2MFA	35.5–41	20	[25]
Dievar	44–46	43	[26]
QRO 90 Supreme	45	15	[27,31]
Vidar Superior	45	30	[28,31]
Bohler W300	50–52	19–28	[29]
Bohler W400	50–52	26–36	[30]

**Table 5:** Comparable hot work steels’ chemical composition.

Element	C (%)	Si (%)	Mn (%)	Cr (%)	Mo (%)	V (%)	Ni (%)	P (%)	S (%)
H13	0.32–0.45	0.8–1.2	0.2–0.5	4.75–5.5	1.1–1.75	0.8–1.2	0.3 max		
Orvar Supreme	0.39	1.0	0.4	5.2	1.4	0.9	-		
33H3MF	0.29–0.36	0.17–0.37	0.5–0.8	2.4–2.8	0.35–0.45	0.2–0.3	0.3 max	0.035 max	0.035 max
WCLV	0.35–0.45	0.8–1.2	0.2–0.5	4.5–5.5	1.2–1.5	0.8–1.1	0.35 max	0.03 max	0.03 max
25H2N4WA	0.21–0.28	0.17–0.37	0.25–0.55	1.35–1.65	-	-	4–4.0	0.03 max	0.025
30HN2MFA	0.26–0.33	0.17–0.37	0.3–0.6	0.6–0.9	0.2–0.3	0.15–0.3	2–2.5	0.03	0.03
Dievar	0.35	0.2	0.5	5	2.3	0.6	-		
Bohler W300	0.38	1.1	0.40	5	1.3	0.4	-		
Bohler W400	0.37	0.2	0.25	5	1.3	0.45	-		
QRO 90 Supreme	0.38	0.3	0.8	2.6	2.3	0.9	-		
Vidar Superior	0.36	0.3	0.3	5	1.3	0.5	-		

**Table 6:** Heat treatment of samples and temperature of testing.

Sample Number	HT 1.	HT 2.	HT 3.	ST (°C)
**1.1** (1∙) **,** **1.2** (1∙∙)	Quenching at 960 °C for 25′	Tempering at 600 °C for 2 h	Tempering at 580 °C for 2 h	+20 ± 2
**1.3** (1∙∙∙) **,** **1.4** (1∙∙∙∙)				−29 ± 2
**2.1** (2∙) **,** **2.2** (2∙∙)	Quenching at 960 °C for 25′	Tempering at 200 °C for 2 h	Tempering at 200 °C for 2 h	+20 ± 2
**2.3** (2∙∙∙) **,** **2.4** (2∙∙∙∙)				−29 ± 2
**3.1** (3∙) **,** **3.2** (3∙∙)	Quenching at 1020 °C for 25′	Tempering at 640 °C for 2 h	Tempering at 600 °C for 2 h	+20 ± 2
**3.3** (3∙∙∙) **,** **3.4** (3∙∙∙∙)				−29 ± 2
**4.1** (4∙) **,** **4.2** (4∙∙)	Tempering at 640 °C for 2 h	Tempering at 600 °C for 2 h	-	+20 ± 2
**4.3** (4∙∙∙) **,** **4.4** (4∙∙∙∙)				−29 ± 2

**Table 7:** Test results: fracture pictures and graphs of exerted energy and load (material after heat treatment).

Sample Number	Graphs	Fracture Area
**1.1** $\left(1_{∙}\right)$	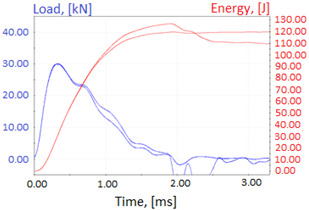	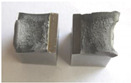
**1.2** (1∙∙)		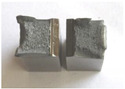
**1.3** (1∙∙∙)	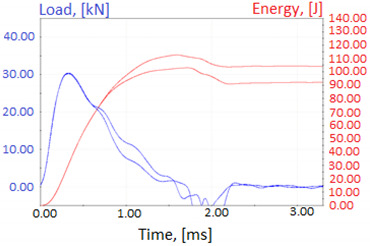	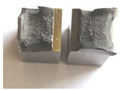
**1.4** (1∙∙∙∙)		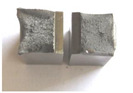
**2.1** (2∙)	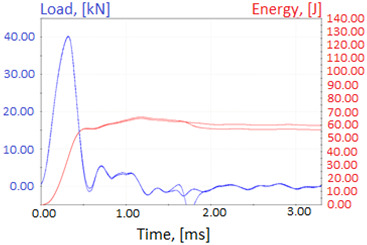	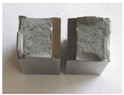
**2.2** (2∙∙)		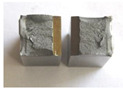
**2.3** (2∙∙∙)	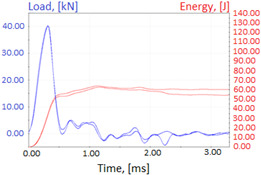	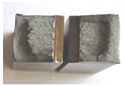
**2.4** (2∙∙∙∙)		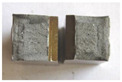
**3.1** (3∙)	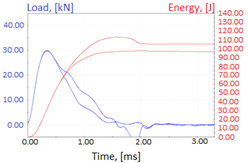	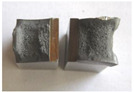
**3.2** (3∙∙)		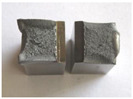
**3.3** (3∙∙∙)	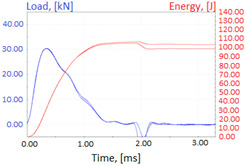	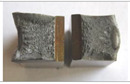
**3.4** (3∙∙∙∙)		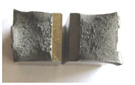
**4.1** (4∙)	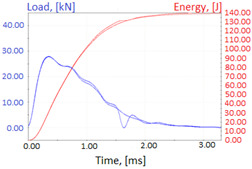	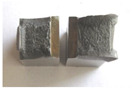
**4.2** (4∙∙)		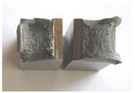
**4.3** (4∙∙∙)	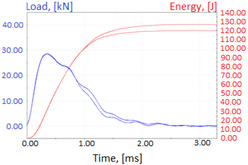	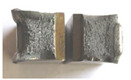
**4.4** (4∙∙∙∙)		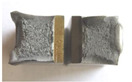

**Table 8:** Experiment results: impact energy, hardness, yield and tensile strength.

Sample Number	Sample Temp. (°C)	Impact Energy KV2 (J)	Mean Impact Energy KV2avg (J)	Mean Hard. (HRC)	Mean Hard. (HV)	YS (MPa)	TS (MPa)
**1.1** (1∙)	+20	126.7	123.2	36.2	356	933	1101
**1.2** (1∙∙)		119.6		35.7	351	918	1084
**1.3** (1∙∙∙)	−29	112.6	107.8	37.5	369	970	1145
**1.4** (1∙∙∙∙)		103.0		36.3	357	936	1104
**2.1** (2∙)	+20	57.2	57.3	48.4	489	1315	1550
**2.2** (2∙∙)		57.4		47.8	482	1295	1526
**2.3** (2∙∙∙)	−29	53.1	58.5	47.7	480	1289	1519
**2.4** (2∙∙∙∙)		63.9		48.5	491	1321	1556
**3.1** (3∙)	+20	97.7	105.4	36.2	356	933	1101
**3.2** (3∙∙)		113.1		36.1	355	933	1101
**3.3** (3∙∙∙)	−29	106.4	105.7	36.1	355	933	1101
**3.4** (3∙∙∙∙)		104.9		36.9	363	953	1124
**4.1** (4∙)	+20	139.2	135.4	33.5	328	852	1006
**4.2** (4∙∙)		131.6		33.6	330	858	1013
**4.3** (4∙∙∙)	−29	126.6	123.2	34.1	336	875	1033
**4.4** (4∙∙∙∙)		119.8		33.7	331	861	1016

## Data Availability

Data is contained within the article.
